# Dalbavancin as long-term suppressive therapy for patients with Gram-positive bacteremia due to an intravascular source—a series of four cases

**DOI:** 10.1007/s15010-020-01526-0

**Published:** 2020-09-23

**Authors:** Florian Hitzenbichler, Arno Mohr, Daniele Camboni, Michaela Simon, Bernd Salzberger, Frank Hanses

**Affiliations:** 1 Department of Infection Prevention and Infectious Diseases, University Hospital Regensburg, Franz-Josef-Strauß-Allee 11, 93053 Regensburg, Germany; 2 Department of Cardiothoracic Surgery, University Hospital Regensburg, Franz-Josef-Strauß-Allee 11, 93053 Regensburg, Germany; 3 Institute for Clinical Microbiology and Hygiene, University Hospital Regensburg, Franz-Josef-Strauß-Allee 11, 93053 Regensburg, Germany; 4 Emergency Department, University Hospital Regensburg, Franz-Josef-Strauß-Allee 11, 93053 Regensburg, Germany

**Keywords:** Dalbavancin, Bacteremia, Intravascular infection, Suppressive therapy, LVAD, Endocarditis

## Abstract

We present four cases with Gram-positive bacteremia (pathogens: MRSA *n* = 1*, Enterococcus* spp. *n* = 3) due to an intravascular source (left ventricular assist device: *n* = 2, transfemoral aortic valve implantation *n* = 1, prosthetic aortic valve: *n* = 1) where no curative treatment was available. These patients received indefinite, chronic suppressive (palliative) therapy with dalbavancin (500 mg weekly or 1000 mg biweekly regimens). Outcomes and clinical characteristics are described; treatment was effective in suppression of bacteremia in all patients over several months (range: 1 to more than 12 months), we observed no relevant side effects.

## Introduction

Long-term suppressive therapy is a last option in patients with chronic bacterial infections, consisting of substances that are well tolerated, have good oral bioavailability and antimicrobial activity. Unfortunately, in some cases not all of these options are available. This might either be due to resistance of the pathogens involved or side effects in patients.

Dalbavancin is a lipoglycopeptide with an exceptional long terminal half-life that was licensed for the treatment of skin and soft tissue infections. It is not licensed for bacteremia, although it is broadly used for this specific indication [1, 2]. It has broad activity against Gram-positive bacteria including methicillin-resistant *Staphylococcus aureus* (MRSA), coagulase-negative staphylococci (CoNS, such as *S. epidermidis*, *S. haemolyticus*) and enterococci (*E. faecalis* and *E. faecium*). Its long half-life of more than 14 days allows weekly or biweekly infusions [3] making it an attractive choice for outpatient antibiotic treatment.

We present a series of four patients with Gram-positive bacteremia due to an intravascular source, where dalbavancin was used as suppressive, indefinite therapy after prior intravenous therapy with different substances.

## Methods

All patients were seen by an infectious diseases specialist before initiating dalbavancin treatment and in all cases oral suppressive therapy was deemed not feasible.

In all four patients a broad interdisciplinary consensus for chronic suppressive therapy was established. Conventional therapy was mainly limited by comorbidities, side effects including allergies and a lack of other (non-antibiotic) options (e.g., surgery and appropriate focus control).

All patients provided informed consent for dalbavancin *off-label* use. The health insurances involved were informed about the *off-label* use of dalbavancin and funding was requested and approved.

Dalbavancin was started after clearance of bacteremia. In all cases dalbavancin was infused by the patients` general practitioner on an out-patient basis.

The isolated organisms (including resistance patterns) are listed in Table 1 together with a short synopsis of the clinical course. Dalbavancin susceptibility testing was not available, but all pathogens involved were susceptible to glycopeptides (vancomycin).

**Table 1:** Characteristics of patients with Dalbavancin suppressive therapy with resistance profiles of the isolated organisms

	Patient 1	Patient 2	Patient 3	Patient 4
Gender/Age	M/81 ys	M/59 ys	M/67 ys	M/80 ys
Isolated pathogen in blood culture	*MRSA*	*E. faecium*	*E. faecalis*	*E. faecalis*
Source of bacteremia	Prosthetic valve endocarditis	LVAD infection	LVAD infection	TAVI endocarditis
Implantation of intravascular prothesis/device	2001	June 2018	January 2018	May 2016
Clinical events of note prior to the onset of the index bacteremia	Admission to another hospital with fever and chills, *MRSA* bacteremia	History of NEM Admission with bowel obstruction/laparotomy/adhesiolysis; *C. diff.* colitis	History of hairy cell leukemia Admission with fever and chills, *E. faecalis* bacteremia (unknown source)	Several admissions with E. faecalis bacteremia, diagnosis of TAVI endocarditis (TEE initially negative)
Index bacteremia (calendar date = d1)	May 2018 (d1)	October 2018 (d1)	May 2019 (d1)	August 2019 (d1)
Initial management of index bacteremia and events up to initiation of dalbavancin	Vancomycin plus rifampicin, later daptomycin plus rifampicin for 6 weeks, cessation of antibiotic therapy on d50	Vancomycin/daptomycin, then switch to dalbavancin	Vancomycin, due to severe penicillin allergy; then chronic suppressive therapy with dalbavancin	Vancomycin and piperacillin (ampicillin/amoxicillin allergy)
Complications/side effects	Readmission on d63 with recurrence of MRSA bacteremia, treatment with vancomycin, then switch to linezolid p.o., followed by dalbavancin	*C. diff. colitis* with acute renal failure, no recurrence despite continuation of dalbavancin, No relevant side effects	Recurrence of *E. faecalis* bacteremia on d161, treatment with vancomycin and then again switch to dalbavancin	Recurrence of bacteremia under oral suppressive therapy with moxifloxacin/rifampicin Mild rash (association to dalbavancin debatable)
Start/end Dalbavancin	d123/d130 (given only twice)	d41/still ongoing	d28/d210	d98/d213
Follow up / outcome	Death on about d135 due to heart failure	Dalbavancin still ongoing	Readmission on d214 with stroke, BC sterile, death due to complications of stroke on d228	Due to general worsening of condition treatment change to best supportive care Death on d223
Dalbavancin regimen	LD 1000 mg, 375 mg weekly	LD 1000 mg, 500 mg weekly	LD 1000 mg, 500 mg weekly	LD 1500 mg, 1000 mg biweekly
Susceptibility testing [MIC (mg/L)]				
Ampicillin	R	R [> 8]	S [< = 2]	S [< = 2]
Amox./Clav	R	R	S	S
Ceftriaxone	R	R	R	R
Pip/Taz	R	R	S	S
Meropenem	R	R	R	R
Ciprofloxacin	R [4]			X [0.5]
Moxifloxacin	R [1]			X [1]
Rifampicin	R [> 1]			R
Cotrimoxazol	R [> 4/76]	R	R	R
Clindamycin	R [1]	R	R	S [1]
Linezolid	S [< 0.5]	S [1]	S [2]	S [1]
Vancomycin	S [1]	S [1]	S [1]	S [< = 0.5]
Daptomycin	R [2]	S [4]	S [4]	
Tetracyclin	I [2]			

The SIR category of antimicrobial agents without MIC value is inferred from an indicator substance or represents intrinsic resistance

*M* male, *ys* years, *LVAD* left ventricular assist device, *TAVI* transcatheter aortic valve implantation, *NEN* neuroendocrine neoplasm, *LD* loading dose, *MIC* minimal inhibitory concentration, *S* susceptible, *R* resistant *X* no EUCAST break-points available, breakpoints were derived from the POET study [12]

## Patient 1

An 81-year-old male patient was transferred to our hospital in June 2018 with methicillin-resistant *S. aureus* (MRSA) bacteremia and a previous history of prosthetic aortic valve replacement in 2001. Transesophageal echocardiography (TEE) showed endocardial vegetations (8 × 5 mm, aortic valve) leading to the diagnosis of prosthetic valve endocarditis (two major DUKE criteria positive).

After initial treatment with vancomycin antibiotic therapy was switched to daptomycin (8 mg/kg) plus rifampicin (2 × 450 mg) and i.v. treatment was continued for 6 weeks. Blood cultures (BC) cleared within several days, no complications arose during the hospital stay.

Twelve days after discharge the patient was readmitted to a different hospital and BC grew MRSA again. The resistance profile showed that linezolid would be the only oral treatment option available. The patient was transferred to our hospital where cardiothoracic surgeons judged major cardiac surgery not feasible because of comorbidities and the patient’s general condition.

The patient was treated with linezolid initially for several days before dalbavancin was administered once weekly (loading dose 1000 mg, followed by 350 mg weekly—dose adjusted for renal insufficiency—GFR CKD-EPI 23 ml/min/1.73qm).

The patient received dalbavancin only twice, before he was readmitted in the beginning of October 2018 with acute decompensated heart failure and transferred to another hospital. He died in November 2018 due to heart failure and kidney failure. There was no clinical suspicion of relapse of MRSA bacteremia, but no BCs were obtained.

## Patient 2

A 59-year-old male patient was admitted to an intensive care unit in our hospital with acute bowel obstruction in September 2018. The patient had a previous history of neuroendocrine neoplasm (NEN). Due to end-stage heart failure (dilated cardiomyopathy (DCM)), a left ventricular assist device (LVAD) had been implanted in June 2018. After laparotomy and adhesiolysis, the patient recovered but then developed an episode of *Clostridioides difficile* infection (CDI) after broad spectrum antibiotic therapy. During treatment with oral vancomycin, the patient developed fever and *Enterococcus faecium* (*E. faecium*) as well as two different species of coagulase-negative staphylococci (*S. epidermidis* and *S. haemolyticus)* were isolated from his blood, all of them in several different BC sets.

The patient was treated with intravenous vancomycin (target trough serum concentration: 15–20 mcg/ml) and later switched to daptomycin (10 mg/kg once daily). Subsequent BCs remained sterile under intravenous therapy. A PET/CT was performed where an infection of the LVAD was suspected. Owing to his general clinical condition and his history of NEN the patient was neither considered a candidate for heart transplantation nor for LVAD exchange. Dalbavancin was offered as salvage therapy. The once weekly infusion was performed by the general practitioner of the patient (loading dose 1000 mg, followed by 500 mg weekly).

He was readmitted several times during the following year (for different reasons: recurrent CDI, acute renal failure; both under dalbavancin therapy, both resolved despite continued dalbavancin infusions). There was no recurrence of bacteremia. A PET/CT performed nearly 1 year after initiating dalbavancin showed evidence of still active LVAD infection and dalbavancin therapy was continued.

At the latest follow-up (more than 1 year of dalbavancin treatment) the patient was in stable condition.

## Patient 3

A 67-year-old male patient was admitted with fever and chills to our hospital in May 2019. He had a history of hairy cell leukemia and toxic cardiomyopathy (gladribine-induced) with LVAD implanted January 2019.

After admission treatment with vancomycin and piperacillin/tazobactam was started. Two days later, two BCs taken at admission grew *Enterococcus faecalis* (*E. faecalis*). I.v. therapy with piperacillin/tazobactam had to be stopped due to an allergic reaction (rash). Vancomycin was continued and lead to clearance of bacteremia.

Again, like in the previously described patient neither heart transplantation nor LVAD exchange (with high surgical risk) was a suitable option for this patient according to an interdisciplinary consensus.

With *E. faecalis* bacteremia in this penicillin-allergic patient dalbavancin chronic suppressive palliative therapy was offered to the patient and started in the end of May 2019 (loading 1000 mg, followed by 500 mg weekly). Since no other focus could be established the LVAD was considered to be the most likely source of bacteremia.

The patients did fine on an outpatient revisit two months later. He was, however, readmitted in October 2019 with sepsis and *E. faecalis* breakthrough bacteremia despite continued dalbavancin therapy (500 mg weekly). He was again treated with vancomycin and cleared bacteremia. Since there were no other options available, dalbavancin was re-administered (loading 1000 mg, followed by 500 mg weekly) and the patient was discharged from our hospital.

He was again re-admitted in the end of November 2019 with (most likely LVAD associated ischemic) stroke. BCs, however, were negative at admission and during his hospital stay. He died in December 2019 due to complications of stroke.

## Patient 4

An 80-year-old male patient was admitted with fever in September 2019. He had a previous history of transcatheter aortic valve implantation (TAVI) due to aortic valve stenosis in May 2016 and several other chronic conditions (chronic renal failure, diabetes, chronic osteomyelitis of the calcaneus, total knee endoprosthesis, early stages of dementia). He was in reduced general condition due to age and comorbidities. BCs grew *E. faecalis.*

During the hospital stay, a thorough work-up was repeated. TEE showed no vegetations on the aortic valve. CT scan, however, revealed septic emboli in kidneys, spleen and brain, leading to the diagnosis of TAVI endocarditis (based on one major and three minor DUKE criteria).

The calcaneus was evaluated by orthopedic surgeons and surgery with amputation was recommended. The patient, however, refused both heart surgery and amputation. Simultaneously, he was deemed not operable owing to his age and comorbidities.

Unfortunately, he had an amoxicillin allergy (rash); amoxicillin re-exposure was tried during the hospital stay, but again, he developed a severe generalized rash.

An oral treatment with moxifloxacin 1 × 400 mg and rifampicin 1 × 600 mg was attempted after several weeks of intravenous therapy (and negative BCs). Although doing well initially under oral therapy, the patient was seen on an outpatient appointment (in October 2019) and relapse of *E. faecalis* bacteremia could be documented. A short treatment course with linezolid over only several days failed due to poor tolerability (nausea).

The patient was readmitted and treated with vancomycin intravenously (and piperacillin, which he tolerated despite his amoxicillin allergy) leading to clearance of bacteremia. After 4 weeks of treatment, the patient was discharged from the hospital (at the beginning of December 2019) with dalbavancin therapy (1500 mg loading, followed by 1000 mg biweekly).

Until March 2020 there was still no clinical sign of bacteremia. A minor rash after the administration of dalbavancin did not lead to discontinuation of the drug. However, his general condition worsened, and the treatment protocol was changed to best supportive care. The patient died in April 2020.

## Discussion

We present four patients with bacteremia caused by Gram-positive cocci due to an intravascular source. All patients had cardiovascular foreign bodies implanted and finally received (or continue to receive) indefinite suppressive therapy with dalbavancin after prior intravenous therapy with other substances.

Patient 1 was treated with dalbavancin only twice, but he could be discharged home and was stable for roughly 4 weeks (2 weeks linezolid and 2 weeks dalbavancin combined), before he was readmitted.

Although dalbavancin therapy was not effective in preventing relapse of infection in patient 3, he did fine for several months after the initial diagnosis of bacteremia and was able to stay at home most of the time after discharge (more than 5 months) which can be considered as a success of suppressive therapy.

The possibility exists, that patient 2 does not have true LVAD infection, but several BC sets were repeatedly positive for different pathogens and PET/CT was suggestive for LVAD infection. On the other hand, antibiotic therapy was never stopped to document recurrence of bacteremia since this was judged be too high of a risk in a patient with severe heart failure. He was also the only patient of our small series, where dalbavancin was started right after the cessation of the previous intravenous therapy (with no oral treatment in between).

Patient 4 also received dalbavancin and the treatment was effective for several months. The only other option for this patient would have been continued vancomycin treatment (due to his renal insufficiency once daily), but implementation would have been much more complicated in an outpatient setting.

These four patients share some unique features:

- 1. All had bacterial infection of a prosthetic device and a curative approach (surgical removal) was not possible.
- 2. There was always broad interdisciplinary consensus that a relapse of bacteremia would inevitably happen if antibiotics were stopped.
- 3. Oral substances for chronic suppressive therapy were not available, not feasible or not effective and an intravenous therapy offered the advantage of higher drug levels in the blood. The long half-life of dalbavancin offers the further advantage of a nearly comfortable dosing interval, even in an outpatient setting.

All isolated pathogens were also susceptible to linezolid. However, problems with myelotoxicity in long-term use are well described in the literature [4] and reflect our own clinical experience. Furthermore, linezolid lacks bactericidal activity of dalbavancin. Outpatient parenteral antimicrobial therapy (OPAT) is not well established in our region and problems arise frequently especially during weekends and on holidays. A once weekly or biweekly infusion is preferable, and implementation is easier.

Patients have been treated with dalbavancin for endocarditis before [1, 5] and a case report describes the (successful) suppressive therapy of MRSA prosthetic valve endocarditis [6]. Dalbavancin was also effective as suppressive therapy in a patient with MRSA LVAD infection (however this patient was not bacteremic) [7]. In a letter to the editor Cicullo et al. describe the case of a patient with MRSA bacteremia and an abdominal aortic prothesis, where dalbavancin was used and resulted in cure of infection [8]. Dalbavancin may also be effective in biofilm-forming infections, as previous in-vitro studies suggest [9]. On the other side dalbavancin treatment in chronic osteomyelitis resulted in poor outcomes (cure rate of less than 40%) [10]. Overall, *off label* use of dalbavancin is common and the substance seems to be an acceptable safe alternative to other intravenous options even in patients with bacteremia.

The ideal dosing regimen for dalbavancin in this setting is still unclear. Several different regimens are published in the literature:

- 1. 1000 mg loading dose, followed by 500 mg weekly [1].
- 2. 1500 mg loading dose, followed by 1000my biweekly [4].
- 3. 1500 mg weekly [7].

Wunsch et al. describe 25 patients with endocarditis and a third of these patients were treated with the same regime as the patients from our series (36% received dalbavancin only once) [1]. Most (63%), but not all patients with endocarditis in the retrospective study of Tobudic et al. were treated with the biweekly regimen [5]. Patients with endocarditis in the Spanish DALBACEN cohort were treated with both dosing regimens also [11].

A completely different approach was applied by Spaziante et al., where serum dalbavancin concentrations were monitored and dosing was managed via drug monitoring [6]. The authors note, however, that the appropriate target concentration for guiding dalbavancin dosing intervals is not yet established.

Although we used the weekly regimen in three of our patients, we do see the benefit in the biweekly approach (meaning less frequent infusions). Drug monitoring would be the optimal approach for monitoring of dalbavancin in our opinion.

Within our limited experience we could not see any relevant side effects of long-term dalbavancin treatment. Two of our patients (No. 2 and 3) received dalbavancin for several months without any documented problems. Patient No. 4 developed a mild rash, which did not lead to discontinuation of the substance. Other studies also documented a low rate of side effects: in one study 5% of patients receiving dalbavancin did experience nausea or rash [10].

Long-term, even indefinite suppressive therapy is a decision on a case-by-case basis and needs to be discussed with the patient and all people involved in patient management (relatives, general practitioner, nursing service, etc.). Long-term side effects of dalbavancin therapy cannot be ruled out with the data of our small case series and patients must be followed closely. Overall tolerance, however, was good in our patients.

## Conclusions

Dalbavancin is an attractive option for outpatient antibiotic therapy including patients in need for a long-term suppressive therapy. Therapy with dalbavancin might be considered in patients with Gram-positive bacteremia due to an intravascular source as a salvage option when other options (such as oral antibiotics) are not available or not feasible. Long-term therapy seems to be well tolerated in these patients with few side effects. Although only once weekly or biweekly application is necessary long-term suppressive therapy still requires a huge effort on several sides (general practitioner, patient, health insurance, nursing service, etc.). Of note, the optimal dosing strategy is still a matter of debate and might depend on several factors. Drug monitoring might possibly be helpful to monitor drug levels and prevent long-term drug accumulation.
